# Supplementary material for: Association of Nutrition in Early Childhood with Body Composition and Leptin in Later Childhood and Early Adulthood
Source: Nutrients. 2021 Sep 18;13(9):3264. doi: 10.3390/nu13093264 (PMC8466313; doi:10.3390/nu13093264)
Supplement: Supplementary file 1 [file nutrients-13-03264-s001.zip › nutrients-1309520-supplementary.pdf]

Supplementary tables

S1: Characteristics and demographics of CIF children who had data available at 9 and 17 years compared with those who did not

Replication analyses

S2: Multiple linear regression models for energy and energy-adjusted macronutrient intakes, using diet collected at 8 months of age to predict body composition and serum leptin concentration at the age of 9 years and body composition at 17 years of age in children enrolled in ALSPAC-CiF (**replication analysis** of Rolland Cachera et al, 1995, 2012 [16, 17])

S3: Multiple linear regression models for energy and energy-adjusted macronutrient intakes, using diet collected at 18 months of age to predict body composition and serum leptin concentration at the age of 9 years and body composition at 17 years of age in children enrolled in ALSPAC-CiF (**replication analysis** of Rolland Cachera et al, 1995, 2012 [16, 17])

Sex-specific extended analyses for diet at 8 months of age

S4: Multiple linear regression models for energy and energy-adjusted macronutrient intakes of **boys**, using diet collected at 8 months of age to predict body composition and serum leptin concentration at the age of 9 years and body composition at 17 years of age in children enrolled in ALSPAC-CiF

S5: Multiple linear regression models for energy and energy-adjusted macronutrient intakes of **boys (plausible reporters only)**, using diet collected at 8 months of age to predict body composition and serum leptin concentration at the age of 9 years and body composition at 17 years of age in children enrolled in ALSPAC-CiF

S6: Multiple linear regression models for energy and energy-adjusted macronutrient intakes of **girls**, using diet collected at 8 months of age to predict body composition and serum leptin concentration at the age of 9 years and body composition at 17 years of age in children enrolled in ALSPAC-CiF

S7: Multiple linear regression models for energy and energy-adjusted macronutrient intakes of **girls (plausible reporters only)**, using diet collected at 8 months of age to predict body composition and serum leptin concentration at the age of 9 years and body composition at 17 years of age in children enrolled in ALSPAC-CiF

Goodness of fit

S8: Goodness of fit for multiple linear regression models for energy and energy-adjusted macronutrient intakes of **boys**, using diet collected at 18 months of age to predict body composition and serum leptin concentration at 9 years of age and body composition at 17 years of age in children enrolled in ALSPAC-CiF (Table 2)

S9: Goodness of fit for multiple linear regression models for energy and energy-adjusted macronutrient intakes of **girls** using diet collected at 18 months of age to predict body composition and serum leptin concentration at 9 years of age and body composition at 17 years of age in children enrolled in ALSPAC-CiF (Table 4)

S1: Characteristics and demographics of CIF children who had data available at 9 and 17 years compared with those who did not

	**Data available at 9 and 17 years**	**Data not available at 9 and 17 years**	**P value (Chi square or t-test)**
Weight at birth (g)	3421±522 (n=561)	3441±535 (n=856)	0.498
Length at birth (cm)	50.5±2.2 (n=490)	50.6±2.1 (n=710)	0.401
BMI at 8 months (kg/m^2)	17.9±1 (n=537)	17.9±1.5 (n=771)	0.886
BMI at 18 months (kg/m^2)	17.1 (n=507)	17.1 (n=649)	0.622
Energy intake at 8 months (kJ)	3368±713 (n=505)	3458±739 (n=672)	0.037
% Energy as fat	35.4±5.1 (n=505)	35.4±5.1 (n=672)	0.950
% Energy protein	13.5±2.6 (n=505)	13.8±2.8 (n=672)	0.112
% Energy carbohydrate	50.9±5.6 (n=505)	50.7±5.7 (n=672)	0.592
Energy intake 18 months (kJ)	4516±894 (n=470)	4702±965 (n=555)	<0.001
% Energy fat	37.1±4.8 (n=470)	37.6±4.7 (n=555)	0.092
% Energy protein	15.4±2.3 (n=470)	15.2±2.2 (n=555)	0.157
% Energy carbohydrate	47.6±5.7 (n=470)	47.3±5.5 (n=555)	0.394
Sex			
Male (%)	47.0 (n=264)	58.5 (n=507)	
Female (%)	53.0 (n=298)	41.5 (n=360)	<0.0001
Breastfeeding			
Never breastfed (%)	14.9 (n=81)	28.4 (n=220)	
Breastfed (%)	85.1 (n=464)	71.6 (n=556)	<0.0001
Gestation (weeks)	39.4±1.8 (n=562)	39.5±1.6 (n=867)	0.426
Mothers age at delivery (years)	29.9±4.5 (n=562)	28.2±4.7 (n=867)	<0.0001
Maternal education			
CSE, O-level or vocational qualifications (%)	50.6 (n=282)	66.7 (n=538)	
A level or degree (%)	49.4 (n=275)	33.3 (n=268)	<0.0001
Maternal BMI (kg/m^2)	23.1±3.8 (n=527)	23.4±4.2 (n=743)	0.216

Values are mean±SD or % (n).

S2: Multiple linear regression models for energy and energy-adjusted macronutrient intakes, using diet collected at 8 months of age to predict body composition and serum leptin concentration at the age of 9 years and body composition at 17 years of age in children enrolled in ALSPAC-CiF (replication analysis of Rolland Cachera et al, 1995, 2012 [16, 17])

	**Models**	**Energy (0.42 MJ)**		**Protein (%E)** [a]		**Carbohydrate (%E)**[a]		**Fat (%E)**					
								**Total**[a]		**SFA**[a]		**MUFA**[a]	
		**B (95% CI)**	**P-value**	**B (95% CI)**	**P-value**	**B (95% CI)**	**P-value**	**B (95% CI)**	**P-value**	**B (95% CI)**	**P-value**	**B (95% CI)**	**P-value**
9 years													
BMI (kg/m^2)	Unadjusted[b] (n=806)	0.1 (-0.02,0.2)	0.094	0.05 (-0.03, 0.13)	0.202	-0.01 (-0.05,0.03)	0.533	0.0 (-0.04, 0.04)	0.948	0.05 (-0.01, 0.1)	0.111	-0.05 (-0.1, 0.05)	0.321
	Adjusted[c] (n=644)	0.1 (0.0, 0.3)	0.044	0.04 (-0.03, 0.12)	0.271	-0.01 (-0.05, 0.03)	0.488	0.0 (-0.04, 0.04)	0.948	0.02 (-0.05, 0.08)	0.623	-0.00 (-0.1, 0.1)	0.957
	Unadjusted[d] (n=783)	0.1 (-0.0, 0.1)	0.083	0.02 (-0.03, 0.06)	0.406	-0.01 (-0.03, 0.02)	0.606	0.0 (-0.02, 0.02)	0.982	0.02 (-0.01, 0.06)	0.197	-0.02 (-0.1, 0.03)	0.410
FFM (kg)	Adjusted[e] (n=628)	0.1 (-0.0, 0.1)	0.138	0.02 (-0.03, 0.06)	0.499	-0.01 (-0.03, 0.01)	0.340	0.01 (-0.02, 0.03)	0.621	0.02 (-0.02, 0.05)	0.405	0.01 (-0.05, 0.1)	0.840
FM (kg)	Unadjusted[d] (n=783)	0.0 (-0.2, 0.2)	0.865	0.1 (-0.05, 0.2)	0.235	-0.03 (-0.1, 0.03)	0.306	0.02 (-0.04, 0.08)	0.557	0.1 (-0.01, 0.18)	0.067	-0.03 (-0.2, 0.1)	0.660
	Adjusted[e] (n=628)	0.1 (-0.1, 0.3)	0.379	0.1 (-0.05, 0.2)	0.260	-0.03 (-0.1, 0.03)	0.309	0.02 (-0.05, 0.08)	0.667	0.03 (-0.07, 0.1)	0.546	0.03 (-0.1, 0.2)	0.721
17 years													
BMI (kg/m^2)	Unadjusted[b] (n=546)	0.0 (-0.2, 0.3)	0.746	0.06 (-0.1, 0.2)	0.411	0.02 (-0.05, 0.08)	0.666	-0.03 (-0.1, 0.04)	0.362	0.01 (-0.1, 0.1)	0.813	-0.1 (-0.3, 0.04)	0.129
	Adjusted[c] (n= 439)	0.0 (-0.2, 0.3)	0.780	0.05 (-0.1, 0.2)	0.537	-0.01 (-0.08, 0.06)	0.064	-0.01 (-0.1, 0.07)	0.856	-0.03 (-0.2, 0.1)	0.673	-0.01 (-0.2, 0.2)	0.898
FFM (kg)	Unadjusted[d] (n=514)	0.1 (-0.1, 0.3)	0.497	0.07 (-0.1, 0.20)	0.358	0.05 (-0.02, 0.11)	0.152	-0.07 (-0.2, -0.01)	0.037	-0.05 (-0.2, 0.06)	0.386	-0.2 (-0.4, -0.1)	0.004
	Adjusted[e] (n=412)	0.10 (-0.1, 0.3)	0.370	0.05 (-0.1, 0.2)	0.523	0.0 (-0.1, 0.1)	0.968	-0.02 (-0.1, 0.06)	0.630	-0.01 (-0.1, 0.1)	0.913	-0.1 (-0.3, 0.1)	0.276
FM (kg)	Unadjusted[d] (n=514)	0.15 (-0.4, 0.7)	0.581	0.2 (-0.2, 0.5)	0.330	0.03 (-0.1, 0.2)	0.741	-0.07 (-0.2, 0.1)	0.428	0.04 (-0.2, 0.3)	0.748	-0.3 (-0.7, 0.1)	0.153
	Adjusted[e] (n=412)	0.14 (-0.4, 0.7)	0.616	0.1 (-0.2, 0.5)	0.504	0.03 (-0.1, 0.2)	0.690	-0.07 (-0.3, 0.1)	0.449	-0.1 (-0.4, 0.2)	0.428	-0.2 (-0.6, 0.3)	0.478
Serum leptin 9 years (ng/ml)	Unadjusted[b] (n=524)	-0.3 (-0.7, 0.2)	0.224	0.1 (-0.2, 0.4)	0.585	-0.01 (0.2, 0.1)	0.852	-0.01 (-0.2, 0.2)	0.950	0.05 (-0.2, 0.3)	0.670	-0.1 (-0.5, 0.2)	0.494
	Adjusted[c] (n=421)	-0.2 (-0.8, 0.3)	0.414	0.1 (-0.2, 0.5)	0.439	-0.04 (-0.2, 0.1)	0.660	0.0 (-0.2, 0.2)	0.998	-0.01 (-0.3, 0.3)	0.934	-0.04 (-0.5, 0.4)	0.840

[a]Nutrients (% energy) adjusted for total energy according to the multivariate nutrient density model. [b]Adjusted for sex. [c]Adjusted for sex, breast feeding, mothers BMI and fathers' social class. [d]Adjusted for sex and height. [e]Adjusted for sex, height, breastfeeding, mothers BMI and fathers' social class.

S3: Multiple linear regression models for energy and energy-adjusted macronutrient intakes, using diet collected at 18 months of age to predict body composition and serum leptin concentration at the age of 9 years and body composition at 17 years of age in children enrolled in ALSPAC-CiF (replication analysis of Rolland Cachera et al, 1995, 2012 [16, 17])

	**Models**	**Energy (0.42 MJ)**		**Protein (%E)** [a]		**Carbohydrate (%E)**[a]		**Fat (%E)**					
								**Total**[a]		**SFA**[a]		**MUFA**[a]	
		**B (95% CI)**	**P-value**	**B (95% CI)**	**P-value**	**B (95% CI)**	**P-value**	**B (95% CI)**	**P-value**	**B (95% CI)**	**P-value**	**B (95% CI)**	**P-value**
9 years													
BMI (kg/m^2)	Unadjusted[b] (n=751)	0.1 (-0.01, 0.2)	0.064	-0.01 (-0.1, 0.1)	0.870	-0.03 (-0.1, 0.01)	0.203	0.04 (0.0, 0.1)	0.105	0.03 (-0.03, 0.1)	0.300	0.2 (0.05, 0.3)	0.007
	Adjusted[c] (n=607)	0.1 (-0.01, 0.2)	0.033	0.03 (-0.1, 0.1)	0.550	-0.03 (-0.1, 0.01)	0.207	0.03 (-0.02, 0.1)	0.221	0.03 (-0.03, 0.1)	0.333	0.2 (0.02, 0.3)	0.022
FFM (kg)	Unadjusted[d] (n=732)	0.1 (0.01, 0.1)	0.024	0.0 (-0.1, 0.1)	0.953	0.01 (-0.01, 0.03)	0.286	-0.02 (-0.04, 0.0)	0.193	-0.03 (-0.1, 0.0)	0.074	-0.04 (-0.1, 0.03)	0.244
	Adjusted[e] (n=596)	0.05 (0.0, 0.1)	0.073	0.0 (-0.03, 0.1)	0.297	0.02 (-0.01, 0.04)	0.223	-0.03 (-0.1, 0.0)	0.046	-0.04 (-0.1, 0.0)	0.037	-0.07 (-0.2, 0.0)	0.063
FM (kg)	Unadjusted[d] (n=732)	0.0 (-0.1, 0.1)	0.827	-0.0 (-0.2, 0.1)	0.618	-0.08 (-0.1, -0.02)	0.006	0.1 (0.06, 0.2)	<0.001	0.1 (0.0, 0.2)	0.067	0.4 (0.2, 0.6)	<0.001
	Adjusted[e] (n=596)	0.0 (-0.1, 0.2)	0.617	0.0 (-0.2, 0.2)	0.964	-0.08 (-0.1, -0.01)	0.02	0.1 (0.03, 0.2)	0.005	0.1 (0.03, 0.2)	0.546	0.4 (0.2, 0.6)	0.001
17 years													
BMI (kg/m^2)	Unadjusted[b] (n=500)	0.1 (-0.1, 0.3)	0.441	-0.2 (-0.3, 0.0)	0.077	-0.03 (-0.1, 0.04)	0.436	0.07 (0.0, 0.2)	0.080	0.1 (-0.02, 0.2)	0.101	0.3 (0.0, 0.5)	0.032
	Adjusted[c] (n= 405)	0.1 (-0.1, 0.3)	0.409	-0.1 (-0.3, 0.1)	0.338	-0.02 (-0.1, 0.05)	0.531	0.05 (0.0, 0.1)	0.239	0.1 (-0.05, 0.2)	0.295	0.3 (0.0, 0.5)	0.044
FFM (kg)	Unadjusted[d] (n=468)	0.1 (-0.1, 0.3)	0.317	-0.1 (-0., 0.1)	0.239	0.06 (-0.0, 0.1)	0.061	-0.1 (-0.2, 0.0)	0.089	-0.1 (-0.2, 0.03)	0.174	-0.2 (-0.4, 0.1)	0.175
	Adjusted[e] (n=378)	0.1 (-0.1, 0.3)	0.383	-0.03 (-0.2, 0.1)	0.754	0.06 (-0.01, 0.1)	0.090	-0.1 (-0.2, 0.0)	0.055	-0.1 (-0.2, 0.02)	0.131	-0.2 (-0.4, 0.1)	0.147
FM (kg)	Unadjusted[d] (n=468)	0.1 (-0.3, 0.6)	0.569	-0.2 (-0.6, 0.2)	0.376	-0.1 (-0.3, 0.04)	0.135	0.2 (0.0, 0.4)	0.031	0.3 (0.05, 0.5)	0.018	0.6 (0.1, 1.1)	0.022
	Adjusted[e] (n=378)	0.2 (-0.3, 0.6)	0.417	-0.05 (-0.5, 0.4)	0.802	-0.1 (-0.3, 0.1)	0.232	0.2 (-0.1, 0.4)	0.136	0.2 (-0.02, 0.5)	0.069	0.6 (0.0, 1.1)	0.055
Serum leptin 9 years (ng/ml)	Unadjusted[b] (n=494)	-0.1 (-0.5, 0.2)	0.442	-0.1 (-0.5, 0.2)	0.501	-0.03 (-0.2, 0.1)	0.708	0.07 (-0.1, 0.3)	0.426	0.1 (-0.2, 0.3)	0.514	0.3 (-0.2, 0.7)	0.304
	Adjusted[c] (n=403)	-0.1 (-0.5, 0.3)	0.677	-0.02 (-0.4, 0.4)	0.918	-0.09 (-0.3, 0.1)	0.299	0.1 (-0.1, 0.4)	0.190	0.16 (-0.1, 0.4)	0.249	0.4 (-0.2, 0.1)	0.151

[a]Nutrients (% energy) adjusted for total energy according to the multivariate nutrient density model. [b]Adjusted for sex. [c]Adjusted for sex, breast feeding, mothers BMI and fathers' social class. [d]Adjusted for sex and height. [e]Adjusted for sex, height, breastfeeding, mothers BMI and fathers' social class.

S4: Multiple linear regression models for energy and energy-adjusted macronutrient intakes of **boys**, using diet collected at 8 months of age to predict body composition and serum leptin concentration at the age of 9 years and body composition at 17 years of age in children enrolled in ALSPAC-CiF

	Models	Energy (0.42 MJ)		Protein (%E)[a]		Carbohydrate (%E)[a]		Fat (%E)					
								Total[a]		SFA[a]		MUFA[a]	
		B (95% CI)	P-value	B (95% CI)	P-value	B (95% CI)	P-value	B (95% CI)	P-value	B (95% CI)	P-value	B (95% CI)	P-value
**9 years**													
BMI (kg/m^2)	Unadjusted[a] (n=428)	0.1 (-0.03, 0.3)	0.116	0.1 (-0.05, 0.2)	0.298	0.0 (-0.1, 0.04)	0.655	0.0 (-0.1, 0.1)	0.975	0.0 (-0.0, 0.1)	0.288	0.0 (-0.2, 0.1)	0.696
	Model 1[b] (n=376)	0.1 (-0.02, 0.3)	0.084	0.0 (-0.1, 0.1)	0.483	0.0 (-0.1, 0.04)	0.800	0.0 (-0.1, 0.1)	0.986	0.0 (-0.1, 0.1)	0.803	0.0 (-0.1, 0.2)	0.646
	Model 2[c] (n=299)	0.1 (-0.1, 0.3)	0.377	0.03 (-0.1, 0.1)	0.649	0.0 (-0.1, 0.05)	0.947	0.0 (-0.1, 0.1)	0.901	0.0 (-0.1, 0.1)	0.780	0.0 (-0.1, 0.2)	0.692
FFM (kg)	Unadjusted[d] (n=417)	0.1 (0.04, 0.2)	0.005	0.04 (0.0, 0.1)	0.203	0.0 (-0.03, 0.03)	0.899	0.0 (-0.05, 0.02)	0.385	0.0 (-0.1, 0.1)	0.977	0.0 (-0.1, 0.04)	0.388
	Model 1[e] (n=367)	0.1 (0.01, 0.2)	0.036	0.02 (0.0, 0.1)	0.551	0.0 (-0.02, 0.04)	0.489	0.0 (-0.05, 0.01)	0.248	0.0 (-0.1, 0.0)	0.473	-0.01 (-0.1, 0.1)	0.773
	Model 2[f] (n=291)	0.1 (-0.04, 0.2)	0.215	0.01 (-0.1, 0.1)	0.896	0.0 (-0.02, 0.05)	0.393	0.0 (-0.1, 0.02)	0.268	0.0 (-0.1, 0.0)	0.426	-0.02 (-0.1, 0.1)	0.736
FM (kg)	Unadjusted[d] (n=417)	0.06 (-0.2, 0.3)	0.646	0.05 (-0.1, 0.2)	0.548	0.0 (-0.1, 0.05)	0.455	0.0 (-0.1, 0.1)	0.528	0.1 (-0.1, 0.2)	0.239	0.0 (-0.2, 0.2)	0.853
	Model 1[e] (n=367)	0.1 (-0.1, 0.4)	0.336	0.05 (-0.1, 0.2)	0.563	0.0 (-0.1, 0.1)	0.479	0.0 (-0.1, 0.1)	0.543	0.0 (-0.1, 0.2)	0.681	0.1 (-0.1, 0.3)	0.413
	Model 2[f] (n=291)	0.1 (-0.2, 0.4)	0.586	0.1 (-0.1, 0.3)	0.441	0.0 (-0.1, 0.1)	0.739	0.0 (-0.1, 0.1)	0.967	0.0 (-0.1, 0.2)	0.834	0.0 (-0.2, 0.3)	0.867
**17 years**													
BMI (kg/m^2)	Unadjusted[a] (n=257)	0.0 (-0.3, 0.3)	0.981	0.03 (-0.2, 0.2)	0.768	0.0 (-0.1, 0.1)	0.850	0.0 (-0.1, 0.1)	0.675	0.0 (-0.2, 0.2)	0.828	0.0 (-0.3, 0.3)	0.999
	Model 1[b] (n=233)	0.0 (-0.3, 0.3)	0.944	-0.1 (-0.3, 0.2)	0.585	0.0 (-0.1, 0.1)	0.668	0.0 (-0.1, 0.1)	0.836	-0.1 (-0.2,0.1)	0.393	0.1 (-0.2, 0.4)	0.445
	Model 2[c] (n=195)	-0.1 (-0.4, 0.3)	0.624	-0.1 (-0.3, 0.1)	0.336	0.0 (-0.1, 0.1)	0.578	0.0 (-0.1, 0.1)	0.861	-0.1 (-0.3,0.1)	0.433	0.1 (-0.2, 0.4)	0.381
FFM (kg)	Unadjusted[d] (n=242)	0.2 (-0.2, 0.5)	0.417	0.1 (-0.1, 0.3)	0.439	0.1 (-0.03, 0.2)	0.174	-0.1 (-0.2, 0.02)	0.054	-0.1 (-0.3 0.1)	0.202	-0.3 (-0.6, -0.1)	0.021
	Model 1[e] (n=218)	0.1 (-0.3, 0.5)	0.541	0.01 (-0.2, 0.3)	0.923	0.1 (-0.02, 0.2)	0.102	-0.1 (-0.3, 0.0)	0.058	-0.2 (-0.4,0.0)	0.070	-0.3 (-0.6, 0.0)	0.089
	Model 2[f] (n=183)	0.2 (-0.3, 0.6)	0.452	0.0 (-0.3, 0.3)	0.948	0.1 (0.0, 0.2)	0.069	-0.2 (-0.3, 0.0)	0.035	-0.2 (-0.4,0.0)	0.124	-0.3 (-0.6, 0.0)	0.082
FM (kg)	Unadjusted[d] (n=242)	0.03 (-0.8, 0.8)	0.950	0.2 (-0.3, 0.7)	0.404	-0.05 (-0.3, 0.2)	0.707	0.0 (-0.3, 0.3)	0.964	0.0 (-0.4, 0.4)	0.935	0.1 (-0.6, 0.7)	0.881

	Model 1[e] (n=218)	0.1 (-0.7, 0.9)	0.840	0.0 (-0.6, 0.5)	0.905	0.0 (-0.2, 0.3)	0.799	-0.03 (-0.3, 0.3)	0.842	-0.2 (-0.6,0.3)	0.461	0.2 (-0.5 0.8)	0.589
	Model 2[f] (n=183)	-0.1 (-1.0, 0.8)	0.770	-0.1 (-0.7, 0.5)	0.681	0.1 (-0.2, 0.3)	0.671	-0.04 (-0.3, 0.3)	0.795	-0.2 (-0.6,0.3)	0.430	0.2 (-0.5, 0.9)	0.583
Serum leptin 9 years (ng/ml)	Unadjusted[a] (n=274)	-0.1 (-0.8, 0.5)	0.677	0.1 (-0.3, 0.5)	0.687	-0.1 (-0.4, 0.1)	0.169	0.2 (-0.1, 0.4)	0.169	0.2 (-0.1, 0.5)	0.211	0.1 (-0.4, 0.6)	0.568
	Model 1[b] (n=243)	-0.05 (-0.7,0.6)	0.890	0.1 (-0.3, 0.6)	0.563	-0.2 (-0.4, 0.1)	0.181	0.2 (-0.1, 0.4)	0.222	0.2 (-0.2, 0.6)	0.295	0.1 (-0.4, 0.7)	0.640
	Model 2[c] (n=204)	0.1 (-0.5, 0.6)	0.828	0.2 (-0.2, 0.6)	0.310	-0.1 (-0.3, 0.05)	0.155	0.1 (-0.1, 0.3)	0.248	0.2 (-0.1, 0.5)	0.164	0.2 (-0.3, 0.6)	0.444

[a]Nutrients (% energy) adjusted for total energy according to the multivariate nutrient density model. [b]Adjusted for total energy, breastfeeding duration, maternal BMI, maternal education, smoking status, birthweight. [c]Model b + paternal BMI. [d]Model a + height. [e]Model b + height. [f]Model b + height and paternal BMI

S5: Multiple linear regression models for energy and energy-adjusted macronutrient intakes of **boys (plausible reporters only)**, using diet collected at 8 months of age to predict body composition and serum leptin concentration at the age of 9 years and body composition at 17 years of age in children enrolled in ALSPAC-CiF

	Models	Energy (0.42 MJ)		Protein (%E) [a]		Carbohydrate (%E)[a]		Fat (%E)					
								Total[a]		SFA[a]		MUFA[a]	
		B (95% CI)	P-value	B (95% CI)	P-value	B (95% CI)	P-value	B (95% CI)	P-value	B (95% CI)	P-value	B (95% CI)	P-value
**9 years**													
BMI (kg/m^2)	Unadjusted[a] (n=378)	0.1 (-0.1, 0.3)	0.443	0.04 (-0.1, 0.2)	0.453	0.0 (-0.1, 0.0)	0.644	0.0 (-0.1, 0.1)	0.922	0.1 (0.0, 0.2)	0.219	-0.1 (-0.2, 0.1)	0.327
	Model 1[b] (n=339)	0.0 (-0.2, 0.2)	0.715	0.1 (-0.1, 0.2)	0.356	0.0 (-0.1, 0.0)	0.647	0.0 (-0.1, 0.1)	0.915	0.0 (-0.1, 0.1)	0.683	-0.1 (-0.2, 0.1)	0.493
	Model 2[c] (n=270)	-0.1 (-0.3, 0.1)	0.396	0.0 (-0.1, 0.1)	0.610	0.0 (-0.1, 0.1)	0.955	0.0 (-0.1, 0.1)	0.780	0.0 (-0.1, 0.1)	0.901	0.0 (-0.2, 0.1)	0.802
FFM (kg)	Unadjusted[d] (n=366)	0.0 (-0.2, 0.1)	0.515	0.0 (-0.1, 0.1)	0.928	0.0 (-0.1, 0.0)	0.298	0.0 (-0.02, 0.1)	0.284	0.1 (0.0, 0.1)	0.038	0.0 (-0.1, 0.1)	0.852
	Model 1[e] (n=330)	-0.1 (-0.2, 0.0)	0.191	0.0 (-0.1, 0.1)	0.996	0.0 (-0.1, 0.0)	0.267	0.0 (-0.02, 0.1)	0.305	0.04 (0.0, 0.1)	0.168	0.0 (-0.1, 0.1)	0.779
	Model 2[f] (n=264)	-0.1 (-0.2, 0.0)	0.120	0.0 (-0.1, 0.1)	0.783	0.0 (-0.1, 0.0)	0.475	0.0 (-0.02, 0.1)	0.418	0.0 (0.0, 0.1)	0.297	0.0 (-0.1, 0.1)	0.605
FM (kg)	Unadjusted[d] (n=366)	0.0 (-0.3, 0.3)	0.400	0.1 (-0.1, 0.3)	0.314	0.0 (-0.1, 0.1)	0.494	0.0 (-0.1, 0.1)	0.816	0.1 (0.0, 0.3)	0.160	-0.1 (-0.3, 0.1)	0.415
	Model 1[e] (n=330)	-0.2 (-0.5, 0.1)	0.286	0.1 (-0.1, 0.3)	0.358	0.0 (-0.1, 0.1)	0.503	0.01 (-0.1, 0.1)	0.884	0.0 (-0.1, 0.2)	0.537	0.0 (-0.3, 0.2)	0.747
	Model 2[f] (n=264)	-0.2 (-0.5, 0.1)	0.260	0.1 (-0.1, 0.3)	0.338	0.0 (-0.1, 0.1)	0.898	-0.02 (-0.1, 0.1)	0.662	0.0 (-0.2, 0.1)	0.779	0.0 (-0.3, 0.2)	0.738
**17 years**													
BMI (kg/m^2)	Unadjusted[a] (n=289)	0.1 (-0.2, 0.4)	0.644	0.1 (-0.1, 0.3)	0.400	0.0 (-0.1, 0.1)	0.673	-0.05 (-0.1, 0.1)	0.390	0.0 (-0.1, 0.2)	0.643	-0.2 (-0.5, 0.0)	0.045
	Model 1[b] (n=256)	-0.1 (-0.4, 0.3)	0.729	0.1 (-0.1, 0.3)	0.513	0.0 (-0.1, 0.1)	0.538	-0.05 (-0.1, 0.1)	0.300	0.0 (-0.2, 0.1)	0.606	-0.2 (-0.4, 0.0)	0.071
	Model 2[c] (n=205)	-0.1 (-0.5, 0.2)	0.481	0.0 (-0.2, 0.2)	0.940	0.0 (-0.1, 0.1)	0.562	-0.03 (-0.1, 0.1)	0.519	0.0 (-0.2, 0.1)	0.830	-0.1 (-0.4, 0.1)	0.384
FFM (kg)	Unadjusted[d] (n=272)	0.0 (-0.2, 0.3)	0.961	0.0 (-0.1, 0.2)	0.740	0.0 (-0.1, 0.1)	0.500	0.0 (-0.1, 0.04)	0.341	0.0 (-0.1, 0.1)	0.854	-0.2 (-0.4, 0.0)	0.089
	Model 1[e] (n=240)	-0.1 (-0.4, 0.2)	0.553	0.1 (-0.1, 0.2)	0.598	0.0 (-0.1, 0.1)	0.475	-0.1 (-0.1, 0.03)	0.254	0.0 (-0.2, 0.1)	0.698	-0.2 (-0.4, 0.0)	0.086
	Model 2[f] (n=192)	-0.2 (-0.5, 0.1)	0.140	-0.1 (-0.3, 0.1)	0.351	0.0 (-0.0, 0.1)	0.269	-0.04 (-0.1, 0.1)	0.407	0.0 (-0.2, 0.1)	0.600	-0.1 (-0.3, 0.1)	0.427
FM (kg)	Unadjusted[d] (n=272)	0.2 (-0.5, 0.9)	0.498	0.1 (-0.4, 0.6)	0.679	0.1 (-0.1, 0.3)	0.382	-0.1 (-0.3, 0.1)	0.289	0.1 (-0.3, 0.4)	0.763	-0.6 (-1.1, 0.0)	0.036

	Model 1[e] (n=240)	-0.1 (-0.8, 0.6)	0.852	0.1 (-0.3, 0.6)	0.603	0.1 (-0.1, 0.3)	0.405	-0.1 (-0.3, 0.1)	0.270	-0.1 (-0.4, 0.3)	0.685	-0.5 (-1.0, 0.0)	0.056
	Model 2[f] (n=192)	-0.1 (-0.9, 0.7)	0.786	-0.1 (-0.6, 0.4)	0.721	0.1 (-0.1, 0.3)	0.403	-0.1 (-0.3, 0.2)	0.473	-0.1 (-0.4, 0.3)	0.779	-0.2 (-0.8, 0.3)	0.412
Serum leptin 9 years (ng/ml)	Unadjusted[a] (n=250)	-0.5 (-1.1, 0.2)	0.177	0.1 (-0.4, 0.5)	0.736	0.1 (-0.1, 0.3)	0.272	-0.2 (-0.4, 0.1)	0.146	-0.1 (-0.5,0.2)	0.517	-0.4 (-0.9, 0.1)	0.120
	Model 1[b] (n=225)	-0.7 (-1.5, 0.1)	0.068	0.1 (-0.4, 0.5)	0.741	0.1 (-0.1, 0.3)	0.328	-0.2 (-0.4, 0.1)	0.163	-0.2 (-0.6,0.2)	0.309	-0.4 (-0.9, 0.2)	0.191
	Model 2[c] (n=181)	-0.7 (-1.6, 0.1)	0.101	0.0 (-0.5, 0.5)	0.989	0.2 (-0.1, 0.4)	0.139	-0.2 (-0.5, 0.0)	0.081	-0.3 (-0.7,0.0)	0.080	-0.4 (-0.9, 0.2)	0.217

[a]Nutrients (% energy) adjusted for total energy according to the multivariate nutrient density model. [b]Adjusted for total energy, breastfeeding duration, maternal BMI, maternal education, smoking status, birthweight. [c]Model b + paternal BMI. [d]Model a + height. [e]Model b + height. [f]Model b + height and paternal BMI

S6: Multiple linear regression models for energy and energy-adjusted macronutrient intakes of **girls**, using diet collected at 8 months of age to predict body composition and serum leptin concentration at the age of 9 years and body composition at 17 years of age in children enrolled in ALSPAC-CiF

	Models	Energy (0.42 MJ)		Protein (%E)[a]		Carbohydrate (%E)[a]		Fat (%E)					
								Total[a]		SFA[a]		MUFA[a]	
		B (95% CI)	P-value	B (95% CI)	P-value	B (95% CI)	P-value	B (95% CI)	P-value	B (95% CI)	P-value	B (95% CI)	P-value
**9 years**													
BMI (kg/m²)	Unadjusted[a] (n=319)	0.3 (0.0, 0.6)	0.047	0.1 (-0.1, 0.2)	0.259	0.0 (-0.1, 0.0)	0.555	0.0 (-0.1, 0.1)	0.892	0.1 (0.0, 0.2)	0.165	-0.1 (-0.3, 0.04)	0.158
	Model 1[b] (n=285)	0.2 (-0.1, 0.5)	0.217	0.1 (-0.03, 0.2)	0.141	0.0 (-0.1, 0.0)	0.452	0.0 (-0.1, 0.1)	0.978	0.1 (-0.1, 0.1)	0.330	-0.1 (-0.2, 0.1)	0.212
	Model 2[c] (n=231)	0.0 (-0.3, 0.3)	0.854	0.1 (-0.1, 0.2)	0.328	-0.02 (-0.1, 0.0)	0.527	0.0 (-0.1, 0.1)	0.885	0.0 (-0.1, 0.1)	0.664	-0.03 (-0.2, 0.1)	0.690
FFM (kg)	Unadjusted[d] (n=308)	0.0 (-0.2, 0.2)	0.962	0.0 (-0.1, 0.1)	0.642	-0.02 (-0.1, 0.0)	0.196	0.0 (-0.02, 0.1)	0.256	0.1 (0.0, 0.1)	0.025	-0.02 (-0.1, 0.1)	0.641
	Model 1[e] (n=276)	0.0 (-0.2, 0.1)	0.672	0.0 (-0.1, 0.1)	0.578	-0.03 (-0.1, 0.0)	0.172	0.0 (-0.02, 0.06)	0.255	0.1 (0.0, 0.1)	0.085	0.0 (-0.1, 0.1)	0.960
	Model 2[f] (n=225)	-0.1 (-0.4, 0.1)	0.169	0.0 (-0.1, 0.1)	0.744	-0.02 (-0.1, 0.0)	0.261	0.02 (-0.02, 0.1)	0.313	0.1 (0.0, 0.1)	0.165	0.0 (-0.1, 0.1)	0.668
FM (kg)	Unadjusted[d] (n=308)	0.1 (-0.3, 0.6)	0.615	0.1 (-0.04, 0.3)	0.134	-0.04 (-0.1, 0.1)	0.408	0.0 (-0.1, 0.1)	0.831	0.1 (0.0, 0.3)	0.108	-0.2 (-0.4, 0.1)	0.174
	Model 1[e] (n=276)	0.0 (-0.4, 0.5)	0.942	0.1 (-0.04, 0.3)	0.126	-0.04 (-0.1, 0.1)	0.359	0.0 (-0.1, 0.1)	0.857	0.1 (-0.1, 0.2)	0.258	-0.1 (-0.4, 0.1)	0.335
	Model 2[f] (n=225)	-0.1 (-0.6, 0.4)	0.596	0.1 (-0.1, 0.3)	0.144	-0.03 (-0.1, 0.1)	0.548	-0.01 (-0.1, 0.1)	0.859	0.0 (-0.2, 0.2)	0.885	-0.1 (-0.3, 0.2)	0.579
**17 years**													
BMI (kg/m²)	Unadjusted[a] (n=244)	0.4 (-0.1, 0.9)	0.150	0.1 (-0.2, 0.3)	0.696	0.0 (-0.1, 0.1)	0.560	-0.1 (-0.2, 0.1)	0.432	0.0 (-0.2, 0.2)	0.865	-0.2 (-0.5, 0.1)	0.109
	Model 1[b] (n=215)	0.3 (-0.2, 0.8)	0.193	0.0 (-0.2, 0.2)	0.941	0.04 (-0.1, 0.1)	0.411	-0.1 (-0.2, 0.1)	0.352	-0.1 (-0.2,0.1)	0.596	-0.2 (-0.5, 0.1)	0.128
	Model 2[c] (n=176)	0.1 (-0.5, 0.6)	0.805	0.0 (-0.3, 0.2)	0.865	0.04 (-0.1, 0.1)	0.486	-0.04 (-0.2, 0.1)	0.467	0.0 (-0.2, 0.2)	0.781	-0.1 (-0.4, 0.2)	0.347
FFM (kg)	Unadjusted[d] (n=228)	0.2 (-0.2, 0.7)	0.254	0.0 (-0.2, 0.2)	0.970	0.04 (-0.04, 0.1)	0.329	-0.1 (-0.1, 0.0)	0.283	0.0 (-0.2, 0.1)	0.776	-0.2 (-0.4, 0.1)	0.124
	Model 1[e] (n=199)	0.1 (-0.3, 0.6)	0.559	0.0 (-0.2, 0.2)	0.896	0.04 (-0.1, 0.1)	0.394	-0.1 (-0.1, 0.1)	0.309	0.0 (-0.2, 0.1)	0.657	-0.2 (-0.4, 0.1)	0.125
	Model 2[f] (n=163)	-0.1 (-0.7, 0.4)	0.595	-0.1 (-0.4, 0.1)	0.250	0.1 (-0.04, 0.2)	0.259	-0.04 (-0.2, 0.1)	0.480	-0.1 (-0.2,0.1)	0.583	-0.1 (-0.3, 0.2)	0.535
FM (kg)	Unadjusted[d] (n=228)	1.0 (-0.1, 2.2)	0.074	0.0 (-0.6, 0.5)	0.920	0.1 (-0.1, 0.3)	0.343	-0.1 (-0.4, 0.1)	0.369	0.1 (-0.4, 0.5)	0.806	-0.5 (-1.1, 0.1)	0.088

	Models	B (95% CI)	P-value	B (95% CI)	P-value	B (95% CI)	P-value	B (95% CI)	P-value	B (95% CI)	P-value	B (95% CI)	P-value
	Model 1[e] (n=199)	0.9 (-0.2, 1.9)	0.110	0.0 (-0.5, 0.5)	0.986	0.1 (-0.1, 0.3)	0.390	-0.1 (-0.3, 0.1)	0.387	0.0 (-0.4, 0.4)	0.952	-0.5 (-1.0, 0.1)	0.092
	Model 2[f] (n=163)	0.5 (-0.8, 1.7)	0.466	-0.1 (-0.7, 0.4)	0.680	0.1 (-0.1, 0.4)	0.350	-0.1 (-0.4, 0.2)	0.411	0.0 (-0.5, 0.4)	0.879	-0.4 (-1.0, 0.3)	0.261
Serum leptin 9 years (ng/ml)	Unadjusted[a] (n=212)	-0.1 (-1.2, 1.0)	0.866	0.0 (-0.5, 0.4)	0.920	0.1 (-0.1, 0.3)	0.312	-0.1 (-0.4, 0.1)	0.296	0.0 (-0.4, 0.4)	0.831	-0.5 (-1.0, 0.1)	0.115
	Model 1[b] (n=190)	-0.2 (-1.4, 0.9)	0.702	0.0 (-0.4, 0.5)	0.899	0.1 (-0.1, 0.3)	0.419	-0.1 (-0.4, 0.1)	0.307	-0.1 (-0.5,0.3)	0.664	-0.5 (-1.1, 0.2)	0.150
	Model 2[c] (n=158)	-0.4 (-1.7, 0.9)	0.540	0.1 (-0.4, 0.6)	0.747	0.2 (-1.0, 0.4)	0.232	-0.2 (-0.5, 0.1)	0.114	-0.3 (-0.7,0.1)	0.162	-0.5 (-1.1, 0.2)	0.151

[a]Nutrients (% energy) adjusted for total energy according to the multivariate nutrient density model. [b]Adjusted for total energy, breastfeeding duration, maternal BMI, maternal education, smoking status, birthweight. [c]Model b + paternal BMI. [d]Model a + height. [e]Model b + height. [f]Model b + height and paternal BMI

S7: Multiple linear regression models for energy and energy-adjusted macronutrient intakes of **girls (plausible reporters only)**, using diet collected at 8 months of age to predict body composition and serum leptin concentration at the age of 9 years and body composition at 17 years of age in children enrolled in ALSPAC-CiF

	Models	Energy (0.42 MJ)		Protein (%E)[a]		Carbohydrate (%E)[a]		Fat (%E)					
								Total[a]		SFA[a]		MUFA[a]	
		B (95% CI)	P-value	B (95% CI)	P-value	B (95% CI)	P-value	B (95% CI)	P-value	B (95% CI)	P-value	B (95% CI)	P-value
**9 years**													
BMI (kg/m^2)	Unadjusted[a] (n=353)	0.2 (-0.01, 0.5)	0.065	0.04 (-0.1, 0.2)	0.461	0.0 (-0.1, 0.02)	0.225	0.04 (0.0, 0.1)	0.274	0.1 (0.0, 0.2)	0.066	0.03 (-0.1, 0.2)	0.662
	Model 1[b] (n=313)	0.2 (-0.1, 0.4)	0.148	0.02 (-0.1, 0.1)	0.781	0.0 (-0.1, 0.03)	0.403	0.0 (-0.03, 0.1)	0.379	0.0 (-0.1, 0.1)	0.392	0.1 (-0.1, 0.3)	0.196
	Model 2[c] (n=250)	0.1 (-0.2, 0.3)	0.530	0.0 (-0.1, 0.1)	0.994	0.0 (-0.1, 0.04)	0.581	0.0 (-0.1, 0.1)	0.503	0.0 (-0.1, 0.1)	0.552	0.1 (-0.1, 0.3)	0.233
FFM (kg)	Unadjusted[d] (n=343)	0.2 (0.1, 0.3)	0.005	0.03 (0.0, 0.1)	0.432	0.0 (-0.04, 0.02)	0.548	0.0 (-0.03, 0.04)	0.830	0.0 (0.0, 0.1)	0.422	0.0 (-0.1, 0.1)	0.860
	Model 1[e] (n=304)	0.1 (0.0, 0.3)	0.064	0.0 (-0.1, 0.1)	0.816	0.0 (-0.03, 0.03)	0.978	0.0 (-0.04, 0.03)	0.815	0.0 (-0.1, 0.1)	0.979	0.03 (-0.1, 0.1)	0.522
	Model 2[f] (n=242)	0.1 (0.0, 0.3)	0.128	0.0 (-0.1, 0.1)	0.803	0.0 (-0.03, 0.04)	0.800	0.0 (-0.1, 0.04)	0.790	0.0 (-0.1, 0.1)	0.903	0.03 (-0.1, 0.1)	0.593
FM (kg)	Unadjusted[d] (n=343)	0.0 (-0.4, 0.4)	0.929	0.1 (-0.1, 0.3)	0.412	-0.1 (-0.1, 0.04)	0.263	0.1 (-0.1, 0.2)	0.337	0.1 (-0.0 ,0.3)	0.116	0.03 (-0.2, 0.3)	0.821
	Model 1[e] (n=304)	0.0 (-0.3, 0.5)	0.884	0.04 (-0.1, 0.2)	0.651	-0.1 (-0.1, 0.04)	0.307	0.1 (-0.1, 0.2)	0.298	0.1 (-0.1, 0.2)	0.464	0.2 (-0.1, 0.4)	0.217
	Model 2[f] (n=242)	0.0 (-0.5, 0.4)	0.873	0.1 (-0.2, 0.3)	0.591	-0.03 (-0.1, 0.1)	0.541	0.03 (-0.1, 0.1)	0.637	0.0 (-0.1, 0.2)	0.719	0.1 (-0.2, 0.4)	0.556
**17 years**													
BMI (kg/m^2)	Unadjusted[a] (n=212)	-0.1 (-0.6, 0.4)	0.705	0.0 (-0.2, 0.3)	0.895	-0.03 (-0.2, 0.1)	0.688	0.03 (-0.1, 0.2)	0.718	0.1 (-0.2, 0.3)	0.634	0.1 (-0.2, 0.4)	0.503

	Model 1[b] (n=193)	-0.2 (-0.7, 0.3)	0.385	-0.1 (-0.3, 0.2)	0.451	0.0 (-0.1, 0.1)	0.981	0.03 (-0.1, 0.2)	0.708	-0.03 (-0.2, 0.2)	0.748	0.3 (-0.1, 0.6)	0.125
	Model 2[c] (n=162)	-0.2 (-0.8, 0.3)	0.387	-0.2 (-0.4, 0.1)	0.275	0.02 (-0.1, 0.2)	0.737	0.01 (-0.1, 0.2)	0.882	-0.1 (-0.3, 0.2)	0.588	0.3 (-0.1, 0.6)	0.143
FFM (kg)	Unadjusted[d] (n=200)	0.1 (-0.4, 0.6)	0.700	0.0 (-0.3, 0.3)	0.962	0.05 (-0.1, 0.2)	0.476	-0.1 (-0.2, 0.1)	0.456	-0.1 (-0.3, 0.2)	0.614	-0.2 (-0.6, 0.1)	0.222
	Model 1[e] (n=181)	0.0 (-0.5, 0.6)	0.967	0.0 (0.3,0.2)	0.796	0.1 (-0.1, 0.2)	0.351	-0.1 (-0.2, 0.1)	0.357	-0.1 (-0.3, 0.1)	0.354	-0.2 (-0.5, 0.2)	0.440
	Model 2[f] (n=152)	0.3 (-0.3, 0.9)	0.344	0.0 (-0.3, 0.3)	0.902	0.1 (-0.05, 0.2)	0.181	-0.1 (-0.3, 0.03)	0.114	-0.1 (-0.4, 0.1)	0.304	-0.3 (-0.7, 0.2)	0.207
FM (kg)	Unadjusted[d] (n=200)	-0.1 (-1.3, 1.1)	0.918	0.3 (-0.3, 0.9)	0.350	-0.1 (-0.4, 0.2)	0.391	0.1 (-0.3, 0.4)	0.635	0.2 (-0.3, 0.7)	0.514	0.2 (-0.6, 1.0)	0.595
	Model 1[e] (n=181)	-0.1 (-1.3, 1.1)	0.905	0.0 (-0.6, 0.6)	0.940	-0.01 (-0.3, 0.3)	0.951	0.02 (-0.3, 0.4)	0.894	-0.1 (-0.6, 0.4)	0.723	0.4 (-0.4, 1.2)	0.350
	Model 2[f] (n=152)	-0.1 (-1.4, 1.3)	0.897	-0.1 (-0.8, 0.6)	0.726	0.03 (-0.3, 0.3)	0.874	0.0 (-0.4, 0.4)	0.995	-0.1 (-0.7, 0.4)	0.606	0.4 (-0.5, 1.3)	0.351
Serum leptin 9 years (ng/ml)	Unadjusted[a] (n=223)	0.0 (-1.1, 1.0)	0.950	0.0 (-0.5, 0.5)	0.996	-0.2 (-0.4, 0.1)	0.212	0.2 (-0.1, 0.5)	0.125	0.2 (-0.2, 0.6)	0.256	0.2 (-0.4, 0.8)	0.541
	Model 1[b] (n=198)	0.0 (-1.1, 1.2)	0.942	0.0 (-0.5, 0.6)	0.906	-0.2 (-0.4, 0.1)	0.206	0.2 (-0.1, 0.5)	0.146	0.2 (-0.2, 0.7)	0.305	0.2 (-0.5, 1.0)	0.549
	Model 2[c] (n=168)	0.1 (-0.8, 1.0)	0.798	0.1 (-0.3, 0.6)	0.502	-0.1 (-0.3, 0.1)	0.222	0.1 (-0.1, 0.4)	0.230	0.2 (-0.2, 0.5)	0.282	0.1 (-0.4, 0.7)	0.668

[a]Nutrients (% energy) adjusted for total energy according to the multivariate nutrient density model. [b]Adjusted for total energy, breastfeeding duration, maternal BMI, maternal education, smoking status, birthweight. [c]Model b + paternal BMI. [d]Model a + height. [e]Model b + height. [f]Model b + height and paternal BMI

S8: Goodness of fit for multiple linear regression models for energy and energy-adjusted macronutrient intakes of **boys**, using diet collected at 18 months of age to predict body composition and serum leptin concentration at 9 years of age and body composition at 17 years of age in children enrolled in ALSPAC-CiF (Table 2)

	Model	Energy/0.42 MJ		Protein (%E)		Carbohydrate (%E)		Fat (%E)					
								Total		SFA		MUFA	
		Adjusted R^2	SE of the estimate	Adjusted R^2	SE of the estimate	Adjusted R^2	SE of the estimate	Adjusted R^2	SE of the estimate	Adjusted R^2	SE of the estimate	Adjusted R^2	SE of the estimate
**9 years**													
BMI, kg/m^2	Model 1[b] (n=360)	0.152	2.601	0.150	2.604	0.160	2.589	0.162	2.584	0.157	2.592	0.168	2.576
	Model 2[c] (n=289)	0.159	2.613	0.156	2.616	0.165	2.602	0.168	2.598	0.163	2.605	0.173	2.591
FFM, kg	Model 1[d] (n=352)	0.723	1.565	0.723	1.566	0.724	1.561	0.726	1.557	0.725	1.559	0.727	1.553
	Model 2[e] (n=283)	0.724	1.552	0.724	1.554	0.726	1.547	0.728	1.541	0.728	1.541	0.730	1.537
FM, kg	Model 1[d] (n=352)	0.341	4.116	0.339	4.122	0.354	4.074	0.361	4.050	0.354	4.074	0.362	4.048
	Model 2[e] (n=283)	0.354	4.186	0.351	4.194	0.363	4.156	0.368	4.139	0.363	4.155	0.366	4.145
**17 years**													
BMI, kg/m^2	Model 1[b] (n=220)	0.139	4.107	0.143	4.097	0.136	4.116	0.139	4.107	0.139	4.107	0.147	4.089
	Model 2[c] (n=185)	0.152	4.084	0.158	4.069	0.149	4.091	0.156	4.075	0.150	4.090	0.168	4.047
FFM, kg	Model 1[d] (n=205)	0.348	4.512	0.346	4.517	0.349	4.508	0.354	4.489	0.345	4.519	0.359	4.472
	Model 2[e] (n=172)	0.388	4.458	0.385	4.469	0.393	4.440	0.399	4.416	0.388	4.456	0.402	4.404
FM, kg	Model 1[d] (n=205)	0.123	9.763	0.121	9.773	0.125	9.753	0.131	9.717	0.136	9.687	0.137	9.684
	Model 2[e] (n=172)	0.138	9.687	0.135	9.706	0.145	9.645	0.154	9.596	0.151	9.616	0.161	9.560

9y Serum leptin, ng/ml	Model 1[b] (n=243)	0.004	9.042	-0.006	8.904	0.007	9.029	0.013	9.000	0.007	9.027	0.010	9.016
	Model 2[c] (n=204)	0.096	6.532	0.001	9.057	0.112	6.475	0.121	6.440	0.123	6.435	0.124	6.432

Prediction interval = 100 – (2 x SEE)%

[a]Nutrients (% energy) adjusted for total energy according to the multivariate nutrient density model. [b]Adjusted for total energy, breastfeeding duration, maternal BMI, maternal education, smoking status, birthweight. [c]Model as shown in footnote b + paternal BMI. [d] Model shown in footnote b + height. [e]Model shown in footnote b + height and paternal BMI

S9: Goodness of fit for multiple linear regression models for energy and energy-adjusted macronutrient intakes of **girls** using diet collected at 18 months of age to predict body composition and serum leptin concentration at 9 years of age and body composition at 17 years of age in children enrolled in ALSPAC-CiF (Table 4)

	Model	Energy/0.42 MJ		Protein (%E)[a]		Carbohydrate (%E)[a]		Fat (%E)					
								Total[a]		SFA[a]		MUFA[a]	
		Adjusted R^2	SE of the estimate	Adjusted R^2	SE of the estimate	Adjusted R^2	SE of the estimate	Adjusted R^2	SE of the estimate	Adjusted R^2	SE of the estimate	Adjusted R^2	SE of the estimate
**9 years**													
BMI, kg/m^2	Model 1[b] (n=360)	0.104	2.759	0.101	2.763	0.104	2.760	0.104	2.760	0.102	2.762	0.101	2.764
	Model 2[c] (n=289)	0.155	2.511	0.156	2.510	0.156	2.509	0.154	2.512	0.155	2.511	0.153	2.514
FFM, kg	Model 1[d] (n=352)	0.711	1.628	0.710	1.631	0.711	1.630	0.711	1.628	0.712	1.627	0.710	1.631
	Model 2[e] (n=283)	0.708	1.616	0.707	1.619	0.708	1.616	0.708	1.616	0.709	1.614	0.707	1.619
FM, kg	Model 1[d] (n=352)	0.215	4.291	0.213	4.296	0.212	4.298	0.212	4.298	0.213	4.296	0.216	4.287
	Model 2[e] (n=283)	0.264	3.854	0.262	3.859	0.262	3.860	0.264	3.856	0.262	3.860	0.276	3.823
**17 years**													
BMI, kg/m^2	Model 1[b] (n=220)	0.197	3.949	0.194	3.956	0.193	3.958	0.194	3.957	0.194	3.957	0.195	3.955
	Model 2[c] (n=185)	0.267	3.805	0.268	3.803	0.263	3.815	0.262	3.816	0.263	3.815	0.268	3.801
FFM, kg	Model 1[d] (n=205)	0.437	3.244	0.440	3.233	0.443	3.225	0.439	3.236	0.445	3.221	0.435	3.248
	Model 2[e] (n=172)	0.465	3.241	0.474	3.216	0.480	3.196	0.473	3.216	0.482	3.189	0.463	3.247
FM, kg	Model 1[d] (n=205)	0.203	8.360	0.199	8.378	0.199	8.380	0.199	8.379	0.200	8.375	0.199	8.379
	Model 2[e] (n=172)	0.252	8.072	0.251	8.074	0.247	8.095	0.247	8.095	0.247	8.096	0.252	8.070

9y Serum leptin, ng/ml	Model 1[b] (n=243)	0.034	8.929	0.029	8.953	0.033	8.935	0.035	8.926	0.033	8.934	0.030	8.951
	Model 2[c] (n=204)	0.036	8.297	0.040	8.279	0.052	8.225	0.048	8.245	0.051	8.229	0.030	8.320

Prediction interval = 100 – (2 x SEE)%

[a]Nutrients (% energy) adjusted for total energy according to the multivariate nutrient density model. [b]Adjusted for total energy, breastfeeding duration, maternal BMI, maternal education, smoking status, birthweight. [c]Model as shown in footnote b + paternal BMI. [d] Model shown in footnote b + height. [e]Model shown in footnote b + height and paternal BMI
